# Pediatric Respiratory Syncytial Virus Hospitalizations and Respiratory Support After the COVID-19 Pandemic

**DOI:** 10.1001/jamanetworkopen.2024.16852

**Published:** 2024-06-13

**Authors:** Zachary A. Winthrop, Jennifer M. Perez, Steven J. Staffa, Michael L. McManus, Melody G. Duvall

**Affiliations:** 1 Department of Anesthesiology, Critical Care and Pain Medicine, Boston Children’s Hospital, Boston, Massachusetts

## Abstract

**Importance:**

After the COVID-19 pandemic, there was a surge of pediatric respiratory syncytial virus (RSV) infections, but national data on hospitalization and intensive care unit use and advanced respiratory support modalities have not been reported.

**Objective:**

To analyze demographics, respiratory support modes, and clinical outcomes of children with RSV infections at tertiary pediatric hospitals from 2017 to 2023.

**Design, Setting, and Participants:**

This cross-sectional study evaluated children from 48 freestanding US children’s hospitals registered in the Pediatric Health Information System (PHIS) database. Patients 5 years or younger with RSV from July 1, 2017, to June 30, 2023, were included. Each season was defined from July 1 to June 30. Prepandemic RSV seasons included 2017 to 2018, 2018 to 2019, and 2019 to 2020. The postpandemic season was delineated as 2022 to 2023.

**Exposure:**

Hospital presentation with RSV infection.

**Main Outcomes and Measures:**

Data on emergency department presentations, hospital or intensive care unit admission and length of stay, demographics, respiratory support use, mortality, and cardiopulmonary resuscitation were analyzed. Postpandemic season data were compared with prepandemic seasonal averages.

**Results:**

A total of 288 816 children aged 5 years or younger (median [IQR] age, 8.9 [3.3-21.5] months; 159 348 [55.2%] male) presented to 48 US children’s hospitals with RSV from July 1, 2017, to June 30, 2023. Respiratory syncytial virus hospital presentations increased from 39 698 before the COVID-19 pandemic to 94 347 after the pandemic (*P* < .001), with 86.7% more hospitalizations than before the pandemic (50 619 vs 27 114; *P* < .001). In 2022 to 2023, children were older (median [IQR] age, 11.3 [4.1-26.6] months vs 6.8 [2.6-16.8] months; *P* < .001) and had fewer comorbidities (17.6% vs 21.8% of hospitalized patients; *P* < .001) than during prepandemic seasons. Advanced respiratory support use increased 70.1% in 2022 to 2023 (9094 vs 5340; *P* < .001), and children requiring high-flow nasal cannula (HFNC) or noninvasive ventilation (NIV) were older than during prepandemic seasons (median [IQR] age for HFNC, 6.9 [2.7-16.0] months vs 4.6 [2.0-11.7] months; for NIV, 6.0 [2.1-16.5] months vs 4.3 [1.9-11.9] months). Comorbid conditions were less frequent after the pandemic across all respiratory support modalities (HFNC, 14.9% vs 19.1%, NIV, 22.0% vs 28.5%, invasive mechanical ventilation, 30.5% vs 38.0%; *P* < .001).

**Conclusions and Relevance:**

This cross-sectional study identified a postpandemic pediatric RSV surge that resulted in markedly increased hospital volumes and advanced respiratory support needs in older children with fewer comorbidities than prepandemic seasons. These clinical trends may inform novel vaccine allocation to reduce the overall burden during future RSV seasons.

## Key Points

**Question:**

How did demographics and clinical outcomes of pediatric patients with respiratory syncytial virus (RSV) infection who required hospitalization and advanced respiratory support differ during the 2022 to 2023 post–COVID-19 pandemic season compared with prepandemic seasons?

**Findings:**

In this cross-sectional study of 288 816 children 5 years or younger from 48 US pediatric hospitals, there was a surge in RSV infections during the 2022 to 2023 postpandemic season, with a 70% increase in children requiring advanced respiratory support. Children requiring respiratory support during the surge were older and had fewer comorbidities than in prepandemic seasons.

**Meaning:**

These findings highlight postpandemic trends in advanced respiratory support use for pediatric RSV infections that can help inform guidelines as new RSV vaccines become more widely available.

## Teaser

This cross-sectional study compares differences in demographics, respiratory support modes, and clinical outcomes for children with respiratory syncytial virus before vs after the COVID-19 pandemic.

## Introduction

Respiratory syncytial virus (RSV) infection is the leading cause of hospitalization of young children due to respiratory complications of bronchiolitis, pneumonia, and apnea.^1,2,3,4,5,6,7^ Before the COVID-19 pandemic, more than 2 million children 5 years or younger in the US required medical care for RSV infections annually, resulting in 80 000 hospitalizations^1,2,8,9^ and as many as 300 deaths.^6,10,11,12,13,14^ Pediatric hospitalizations for RSV infections decreased markedly during the early COVID-19 pandemic as public health strategies disrupted typical virus circulation patterns, resulting in many children evading RSV exposure in the first years of life.^15,16,17^ Pediatric RSV infections resurged in 2022 to 2023 after the removal of social distancing and masking,^18^ resulting in a substantially increased number of hospitalizations and intensive care unit (ICU) admissions.^19,20^ Emerging data suggest that altered virus seasonality and absent early-life virus exposure during the pandemic have shifted the pediatric RSV and bronchiolitis demographic, with older children requiring hospitalization compared with prepandemic seasons.^19,21,22,23^

Palivizumab, a monoclonal antibody vaccine against RSV F protein, has been the only available licensed product for RSV prophylaxis, but it is limited to use in high-risk infants, is prohibitively expensive,^24,25,26^ and reduces hospitalizations but not mortality.^27,28^ New opportunities for RSV prevention in infants and young children are now available for the first time in decades.^29^ In 2023, the US Food and Drug Administration approved an RSV vaccine for pregnant women^30^ and a long-acting RSV-neutralizing monoclonal antibody for children, both of which show promise for protecting young children against medically attended RSV lower respiratory tract infection.^31^ Historically, most RSV-infected children requiring hospitalization are previously healthy,^1,4,32^ and children with medical comorbidities, including prematurity, heart disease, and chronic lung disease, have increased morbidity and mortality.^6,7,33^ Severe pediatric RSV infection is managed with advanced respiratory support modes, including high-flow nasal cannula (HFNC), noninvasive ventilation (NIV), or invasive mechanical ventilation (IMV) and, less frequently, high-frequency ventilation (HFV), extracorporeal membrane oxygenation (ECMO), and inhaled nitric oxide (iNO).^4^ During the past decade, technological advances have facilitated increased use of HFNC and NIV for bronchiolitis,^34,35,36^ with comparably stable IMV use.^37^ More data are needed to better understand the shifting landscape of pediatric RSV infection in the postpandemic era to identify populations that develop severe RSV infection and may benefit from newly available prophylactic strategies. This analysis compares the clinical outcomes of children with RSV 5 years or younger before, during, and after the COVID-19 pandemic. We hypothesized that the postpandemic 2022 to 2023 RSV season would identify an older, previously healthy population requiring hospitalization, intensive care, and advanced modes of respiratory support in altered numbers and proportions than prepandemic RSV seasons.

## Methods

### Study Design

This retrospective cross-sectional study evaluated patients from 48 US children’s hospitals participating in Pediatric Health Information System (PHIS), a quality-controlled, anonymized, administrative database that contains clinical and resource utilization data from pediatric hospitals in the US that are affiliated with the Children’s Hospital Association.^38^ Participating hospitals provide discharge and encounter data, including *International Statistical Classification of Diseases and Related Health Problems, Tenth Revision* (*ICD-10*) diagnostic and procedure codes, billing data, and administrative data.^38^ This study was granted exemption status by the Boston Children’s Hospital Institutional Review Board because it is secondary research. Informed consent was waived because deidentified data were used. This study follows the Strengthening the Reporting of Observational Studies in Epidemiology (STROBE) reporting guideline.^39^

### Patient Selection

The PHIS database was queried to identify patients 5 years or younger presenting to hospitals with RSV infection between July 1, 2017, and June 30, 2023. This patient population was selected to account for demographic shifts in age distribution observed clinically in the postpandemic season. Hospitals were included if data were available for all study period quarters. Patients were identified by principal admission diagnoses using *International Statistical Classification of Diseases, Tenth Revision, Clinical Modification* (*ICD-10-CM*) codes J20.5, J21.0, J12.1, and B97.4 for RSV infection.

### Data Extraction

Demographic variables collected for each patient encounter during the study period included age (grouped as 0-12 months, 12-24 months, and ≥24 months), sex, race and ethnicity, and Child Opportunity Index 2.0 score. Clinical characteristics included level of service (emergency department [ED], inpatient, ICU, or neonatal ICU), hospital admission and discharge dates, and presence of complex chronic conditions as identified by the “complex chronic condition” flag in the PHIS database.^40^ Race and ethnicity data were included based on previous data highlighting the exacerbation of racial and ethnic disparities in health care access during and after the COVID-19 pandemic, and these data were obtained via the PHIS database.^41^ Outcomes assessed included hospital and ICU length of stay (LOS), use of respiratory support, cardiopulmonary resuscitation, ECMO, iNO, and in-hospital mortality. Mode of respiratory support was queried using the Clinical Transaction Classification codes (Truven Health Analytics)^42^ for HFNC, NIV, IMV, and HFV. Noninvasive ventilation included continuous positive airway pressure, bilevel positive airway pressure, noninvasive positive pressure ventilation, and intermittent positive pressure breathing. Children’s Hospital Association remapped PHIS HFNC codes in March 2023 and retroactively applied this coding to PHIS encounters from January 2016 onward, addressing previous limitations to including HFNC data.^43^ The HFNC data were available from 33 of the 48 study hospitals. The RSV seasons were defined from July 1 to June 30 to account for winter-predominant virus seasonality. The prepandemic period was defined as 3 RSV seasons preceding the COVID-19 pandemic: 2017 to 2018, 2018 to 2019, and 2019 to 2020. The postpandemic RSV season was defined as 2022 to 2023.

### Statistical Analysis

Data are reported as medians (IQRs) for continuous variables and numbers (percentages) for categorical variables. The Kruskal-Wallis test was used for continuous variables, and the Pearson χ^2^ test or Cochran-Armitage χ^2^ test for trend was used for categorical variables to assess statistical differences. Comparisons between the prepandemic and 2022 to 2023 postpandemic RSV seasons were performed using the Wilcoxon rank sum test. Totaled hospital-, ICU-, and respiratory support–days (HFNC, NIV, IMV, and ECMO) were calculated using Clinical Transaction Classification codes. A 2-tailed *P* < .05 was considered statistically significant. All analyses were performed with Stata software, version 17.1 (StataCorp LLC).

## Results

### The 2022 to 2023 Pediatric RSV Surge

**Figure 1.**
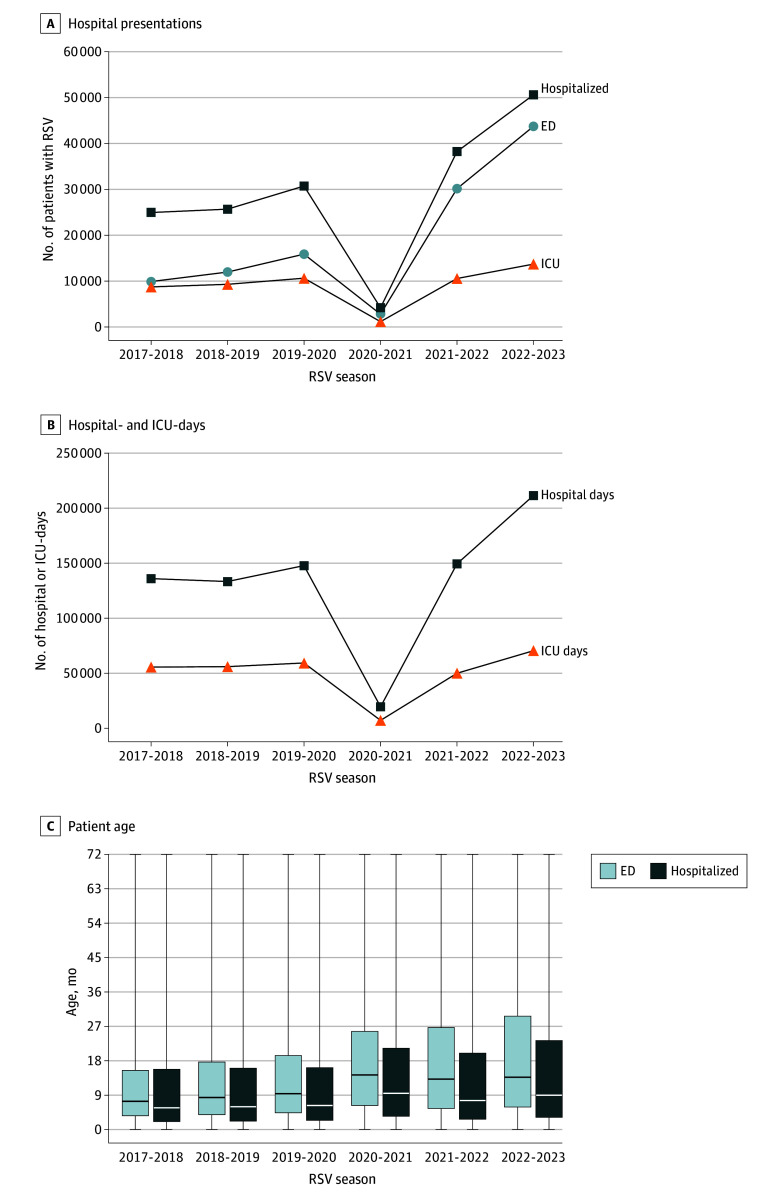
Hospital Presentations, Hospital- and Intensive Care Unit (ICU)–Days, and Patient Age by Respiratory Syncytial Virus (RSV) Season The x-axis denotes RSV seasons, defined as July 1 to June 30. Boxes indicate IQRs, with the horizontal line indicating the mean, and error bars indicate minimum and maximum values.

From July 1, 2017, to June 30, 2023, a total of 288 816 pediatric patients 5 years or younger presented to 48 US children’s hospitals for RSV infections (Table). The median (IQR) age was 8.9 (3.3-21.5) months; 159 348 (55.2%) were male and 129 403 (44.8%) were female; 8076 (2.8%) were Asian or Pacific Islander, 73 220 (25.4%) were Hispanic, 5323 (1.8%) were multiracial, 54 551 (18.9%) were non-Hispanic Black, 121 953 (42.2%) were non-Hispanic White, and 25 693 (8.9%) were of other or unknown race and ethnicity (patients denoted as “other” in the PHIS database and those who declined to provide race and ethnicity information) (eTable 1 in Supplement 1). Pediatric RSV infections decreased markedly in the early COVID-19 pandemic, with 82.0% fewer presentations to hospitals in 2020 to 2021 compared with the 2017 to 2020 prepandemic annual mean (6985 vs 39 698; *P* < .001) (Figure 1A and Table). There was a significant resurgence of RSV infections in 2022 to 2023 compared with prepandemic seasons, with a 2.4-fold increase in the number of children presenting to the hospital for care (94 347 vs 39 698; *P* < .001) (Table). During the 2022 to 2023 postpandemic season, there was a 3.5-fold increase in RSV presentations to EDs that did not require hospital admission (43 728 vs 12 584; *P* < .001) (Figure 1A), an 86.7% increase in RSV hospital admissions (50 619 vs 27 114; *P* < .001) (Figure 1A), and a 43.5% increase in ICU admissions (13 696 vs 9546; *P* < .001) (Figure 1A and Table). There were substantially more total hospital- (211 608 vs 139 079) and ICU-days (70 552 vs 57 121) for RSV during the 2022 to 2023 postpandemic season compared with the prepandemic seasonal mean (Figure 1B).

**Table.** Patient Characteristics

Characteristic	Overall (N = 288 816)	Prepandemic (2017-2020 mean) (n = 119 095; mean n = 39 698)^a^	Postpandemic (2022-2023 season) (n = 94 347)	*P* value^b^
Age, median (IQR), mo				
ED and inpatient	8.9 (3.3-21.5)	6.8 (2.6-16.8)	11.3 (4.1-26.6)	<.001
ED	11.5 (5-24.8)	8.5 (3.9-17.9)	13.7 (5.8-29.8)	
Inpatient	7.2 (2.5-19.0)	6 (2.2-16.1)	9 (3.1-23.4)	
Age group, mo				
0-12	167 417 (58.0)	25 871 (65.2)	48 775 (51.7)	<.001
12-24	57 942 (20.1)	7495 (18.9)	19 177 (20.3)	
≥24-60	63 457 (22.0)	1900 (16.0)	26 395 (28.0)	
Sex				
Female	129 403 (44.8)	17 658 (44.5)	42 461 (45.0)	.002
Male	159 348 (55.2)	22 024 (55.5)	51 870 (55.0)	
Unknown	65 (0.02)	16 (0.04)	16 (0.02)	
Race and ethnicity				
Asian or Pacific Islander	8076 (2.8)	1056 (2.7)	3176 (3.4)	<.001
Hispanic	73 220 (25.4)	9552 (24.1)	25 162 (26.7)	
Multiracial	5323 (1.8)	559 (1.4)	2243 (2.4)	
Non-Hispanic Black	54 551 (18.9)	7157 (18.0)	17 210 (18.2)	
Non-Hispanic White	121 953 (42.2)	17 548 (44.2)	38 211 (40.5)	
Other and unknown^c^	25 693 (8.9)	3826 (9.6)	8345 (8.9)	
Child Opportunity Index score, median (IQR)	45 (21-72) [n = 288 324]	44 (20-70) [n = 118 853]	47 (22-74) [n = 94 214]	<.001
Level of service				
Emergency department	114 421 (39.6)	12 584 (31.7)	43 728 (46.4)	<.001
Inpatient	174 395 (60.4)	27 114 (68.3)	50 619 (53.6)	
ICU and NICU (% of inpatients)	54 053/174 395 (30.9)	9546/27 114 (35.2)	13 696/50 619 (27.1)	
Complex chronic conditions (% of inpatients and observation patients)	34 209/174 395 (19.6)	5910/27 114 (21.8)	8925/50 619 (17.6)	<.001
Length of inpatient stay, median (IQR), d				
Hospital stay	3 (1-5)	3 (2-5)	3 (1-4)	<.001
ICU and NICU stay	3 (2-5)	3 (2-6)	3 (2-5)	
Total hospital- and ICU-days, No.				
Hospital-days	798 095	139 079	211 608	NA
ICU-days	299 283	57 121	70 552	
Respiratory support (% of inpatients)				
HFNC^d^	19 002/136 497 (13.9)	2945/20 787 (14.2)	5752/39 626 (14.5)	.30
NIV	7800/174 395 (4.5)	1413/27 114 (5.2)	2079/50 619 (4.1)	<.001
IMV	4982/174 395 (2.9)	982/27 114 (3.6)	1263/50 619 (2.5)	<.001
HFV	73/174 395 (0.04)	19/27 114 (0.07)	7/50 619 (0.01)	<.001
ECMO	48/174 395 (0.03)	9/27 114 (0.03)	13/50 619 (0.03)	.30
iNO	383/174 395 (0.22)	98/27 114 (0.36)	52/50 619 (0.10)	<.001
Total respiratory support–days				
HFNC	54 667	8876	15 923	NA
NIV	24 224	4612	6114	
IMV	28 000	5912	6104	
ECMO	456	83	129	
Mortality (% of inpatient and ICU patients)				
Inpatient and observation	294/174 395 (0.17)	53/27 114 (0.2)	68/50 619 (0.13)	.009
ICU and NICU	282/54 053 (0.52)	51/9546 (0.53)	64/13 696 (0.47)	.33
CPR (% of inpatient)				
Inpatient and observation	353/174 395 (0.20)	77/27 114 (0.28)	65/50 619 (0.13)	<.001
ICU and NICU	279/54 053 (0.52)	64/9546 (0.67)	44/13 696 (0.32)	<.001

Abbreviations: CPR, cardiopulmonary resuscitation; ECMO, extracorporeal membrane oxygenation; HFNC, high-flow nasal cannula; HFV, high-frequency ventilation; ICU, intensive care unit; IMV, invasive mechanical ventilation; iNO, inhaled nitric oxide; NA, not applicable; NICU, neonatal intensive care unit; NIV, noninvasive ventilation.

^a^ Prepandemic data are reported as means. Percentages are reported as the frequency among all patients unless otherwise indicated.

^b^ Kruskal-Wallis test was used for continuous variables and Pearson χ^2^ test and Cochran-Armitage χ^2^ test for trend for categorical variables. Comparisons between prepandemic and postpandemic seasons were performed using the Wilcoxon rank sum test.

^c^ Includes patients denoted as “other” in the Pediatric Health Information System database and those who declined to provide race and ethnicity information.

^d^ HFNC data were only available from 33 of the 48 study hospitals; therefore, the denominator is different from other respiratory support types. Proportions were calculated based on the percentage of inpatients admitted to those 33 hospitals.

### Patient Population Demographics

Children with RSV in the 2022 to 2023 postpandemic season were significantly older than in prepandemic seasons (median [IQR], 11.3 [4.1-26.6] vs 6.8 [2.6-16.8] months; *P* < .001) (Figure 1C, Table). Children with RSV presenting to the ED who did not require hospitalization were notably older during the 2022 to 2023 postpandemic season than in prepandemic seasons (median [IQR], 13.7 [5.8-29.8] vs 8.5 [3.9-17.9] months; *P* < .001) (Figure 1C and Table). Hospitalized children with RSV in the 2022 to 2023 postpandemic season were also older than in prepandemic seasons (median [IQR], 9 [3.1-23.4] vs 6 [2.2-16.1] months; *P* < .001) (Figure 1C). In all RSV seasons, patients who required hospitalization were younger than those who were discharged home from the ED (median [IQR], 7.2 [2.5-19.0] vs 11.5 [5-24.8] months; *P* < .001) (Figure 1C). Notably, more previously healthy children required hospitalization during the 2022 to 2023 postpandemic season than prepandemic, with a lower proportion of patients having a preexisting comorbid condition (17.6% vs 21.8%; *P* < .001) (Table).

### Hospital Course

**Figure 2.**
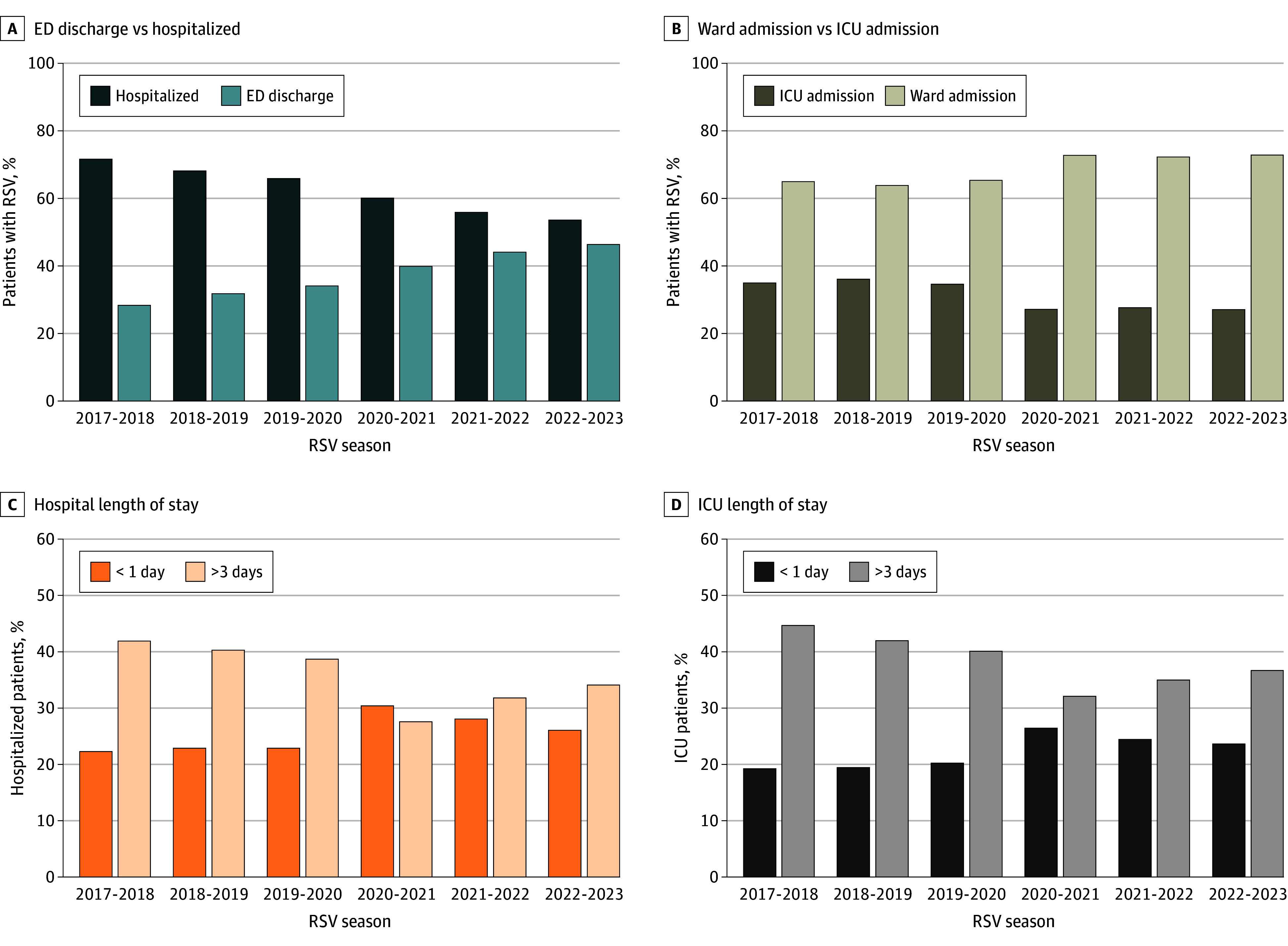
Hospital and Intensive Care Unit (ICU) Admissions and Length of Stay by Respiratory Syncytial Virus (RSV) Season The x-axis denotes RSV seasons, defined as July 1 to June 30. ED indicates emergency department.

Although the total number of patients admitted to the hospital and ICU during the 2022 to 2023 postpandemic season was higher than in prepandemic seasons, a lower proportion of patients with RSV presenting for care required hospitalization (53.6% vs 68.3% before the pandemic; *P* < .001) (Figure 2A and Table). Of patients requiring hospitalization, a lower proportion required ICU admission than in prepandemic seasons (27.1% vs 35.2%; *P* < .001) (Figure 2B). Hospital LOS was shorter during the 2022 to 2023 postpandemic season than in prepandemic seasons, with more patients with a 1-day LOS (26.1% vs 22.7%; *P* < .001) (Figure 2C) and fewer with a greater than 3-day LOS (34.1% vs 40.3%; *P* < .001) (Figure 2C). Similarly, ICU stays were shorter during the postpandemic season with fewer patients requiring ICU admission greater than 3 days (36.8% vs 42.2%; *P* < .001) (Figure 2D and Table). Although the total number of deaths during hospitalization for RSV increased by 28.3% in the 2022 to 2023 postpandemic season compared with the prepandemic mean (68 vs 53; *P* = .007) (eFigure 1A and eTable 1 in Supplement 1), the mortality rate was lower in the total hospitalized population (0.13% vs 0.20%; *P* = .007) (eFigure 1A and eTable 1 in Supplement 1). Of note, the mortality rate among ICU patients was not different in the prepandemic and postpandemic RSV seasons (0.47% vs 0.53%) (eFigure 1A and eTable 1 in Supplement 1). Cardiopulmonary resuscitation (CPR) is a rare event in the pediatric RSV population, and a lower proportion of ICU patients required CPR during the 2022 to 2023 postpandemic season (0.32% vs 0.67%; *P* < .001) (eFigure 1B in Supplement 1).

### Respiratory Support Modes

**Figure 3.**
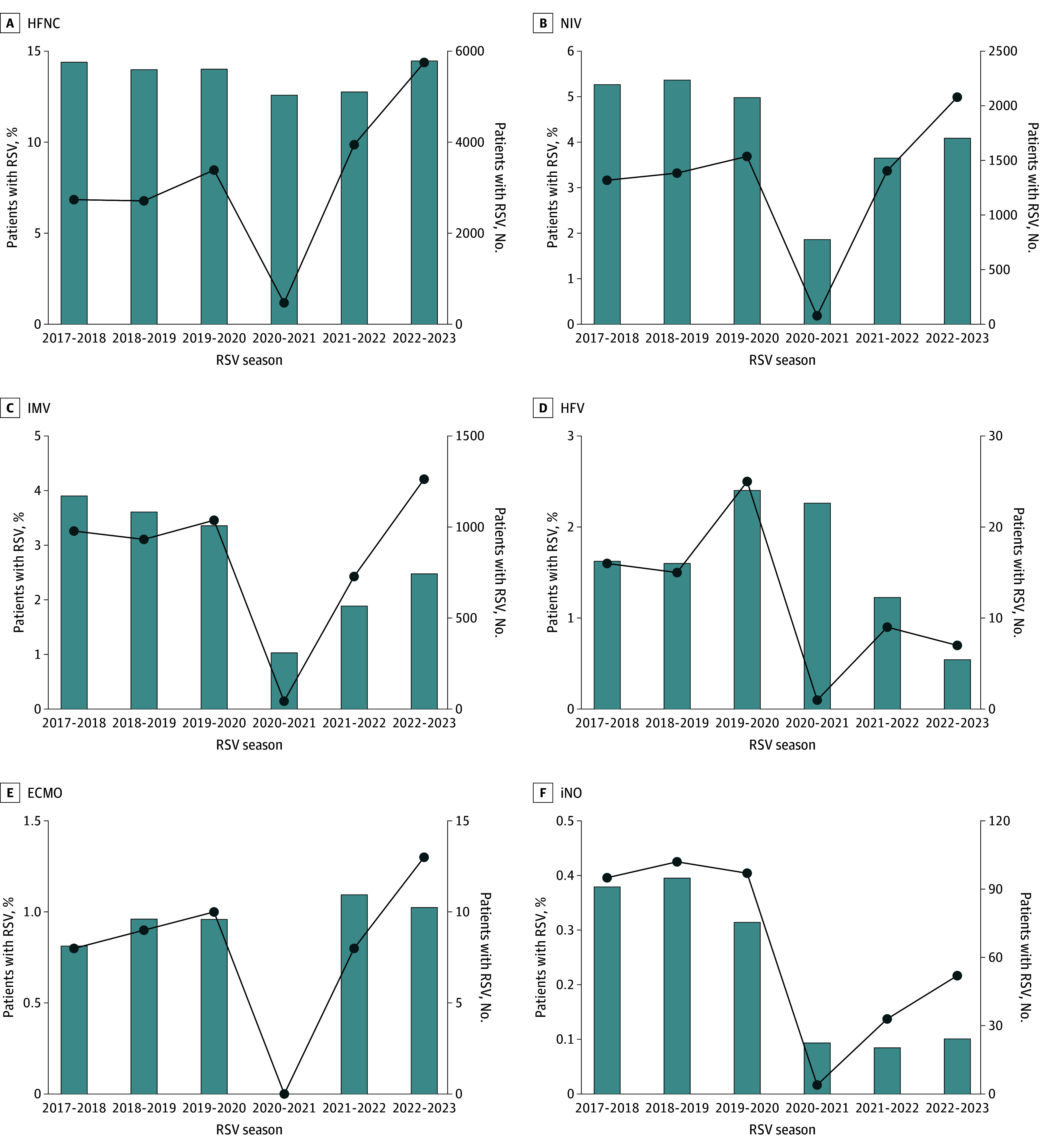
Respiratory Support Mode Use by Respiratory Syncytial Virus (RSV) Season Total number and proportional respiratory support requirements for patients receiving high-flow nasal cannula (HFNC) (A), noninvasive ventilation (NIV) (B), invasive mechanical ventilation (IMV) (C), high-frequency ventilation (HFV) (D), extracorporeal membrane oxygenation (ECMO) (E), and inhaled nitric oxide (iNO) (F). The x-axis denotes RSV seasons, defined as July 1 to June 30. Bars (left y-axis) indicate the proportion of total hospitalized patients requiring the respective respiratory support mode. Circles with connecting line (right y-axis) indicate the total number of patients requiring the respective respiratory support mode. Each panel has a different y-axis scale to show trends.

Across all seasons, 21.6% of hospitalized patients required advanced respiratory support. The total number of hospitalized patients requiring advanced respiratory support (HFNC, NIV, IMV, HFV, ECMO, or iNO) was 70.1% higher during the 2022 to 2023 postpandemic season compared with the prepandemic seasons (9094 vs 5340; *P* < .001) (eFigure 2A and eTable 1 in Supplement 1). The largest increase was in HFNC use, which nearly doubled during the 2022 to 2023 postpandemic season compared with prepandemic seasons (5752 vs 2945; *P* < .001) (Figure 3A and Table). In addition, NIV support increased 47.1% (2079 vs 1413; *P* < .001) (Figure 3B and Table), and IMV use increased 28.6% (1263 vs 982; *P* < .001) (Figure 3C and Table). Of note, the proportion of patients supported with HFNC was not different than in prepandemic seasons (14.5% vs 14.2%) (Figure 3A), whereas a lower percentage were managed with NIV (4.1% vs 5.2%; *P* < .001) (Figure 3B) or IMV (2.5% vs 3.6%; *P* < .001) (Figure 3C). Among patients receiving IMV, the total number and relative proportion supported with HFV was lower in the 2022 to 2023 postpandemic season (7 vs 19 [*P* < .001] and 0.6% vs 1.9% [*P* = .009]) (Figure 3D). Use of ECMO for RSV is rare and did not differ in the 2022 to 2023 season (Figure 3E). Use of iNO was significantly lower in the 2022 to 2023 postpandemic season (52 [0.1%] vs 98 [0.4%]; *P* < .001) (Figure 3F).

**Figure 4.**
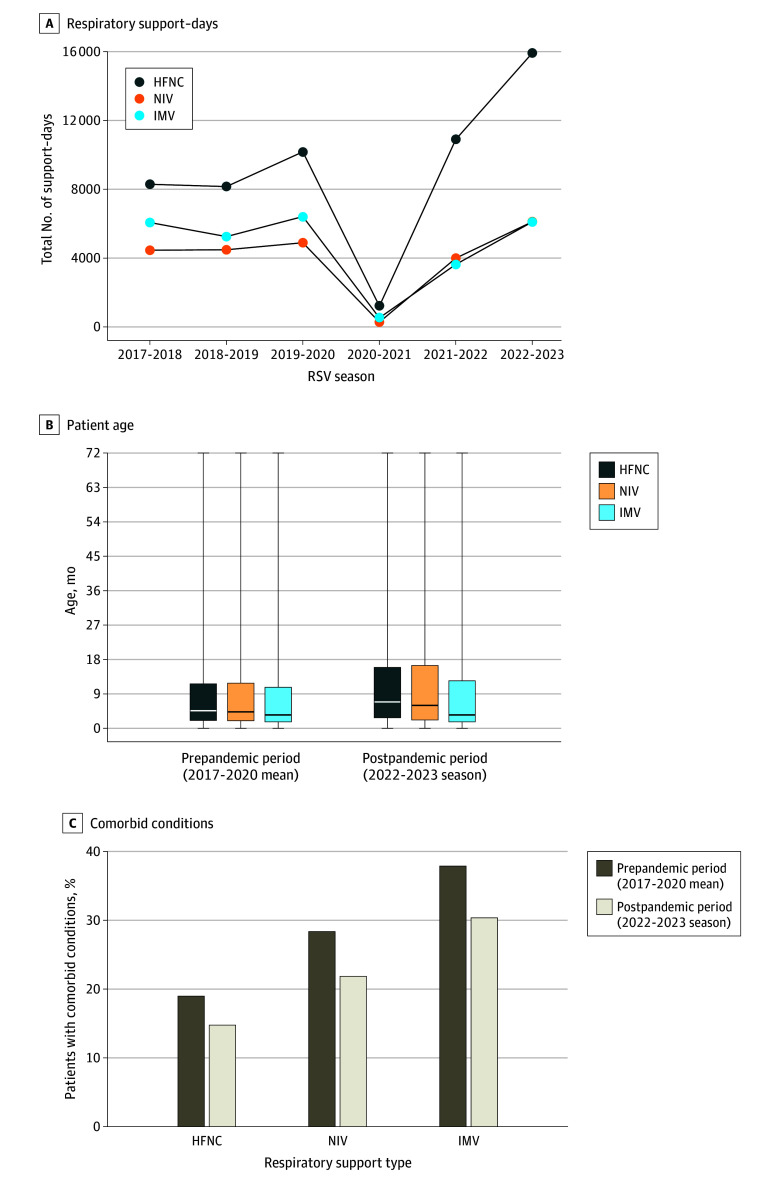
Respiratory Support–Days, Age, and Comorbidities by Respiratory Syncytial Virus (RSV) Season A, Total support-days for high-flow nasal cannula (HFNC), noninvasive ventilation (NIV), and invasive mechanical ventilation (IMV) are shown for each RSV season. The x-axis denotes RSV seasons, defined as July 1 to June 30. B, Age for HFNC, NIV, and IMV inpatient populations during prepandemic vs postpandemic seasons are shown. Boxes indicate IQRs, with the horizontal line indicating the mean, and error bars indicate minimum and maximum values. C, Proportions of patients with comorbid conditions for each respiratory support type for prepandemic vs postpandemic inpatient populations are shown.

There were substantially more advanced respiratory support–days during the 2022 to 2023 postpandemic season, largely driven by HFNC-days, which increased 79.6% from prepandemic seasons (15 923 vs 8876) (Figure 4A and Table). In contrast, total IMV-days were similar (6104 vs 5912) (Figure 4A). Although there were more total respiratory support–days during the 2022 to 2023 postpandemic season, a lower proportion of patients required prolonged support of greater than 3 days with HFNC (24.3% vs 29.2%), NIV (26.8% vs 30.9%), or IMV (42.5% vs 53.6%; *P* < .001) (eFigure 2B-D and eTable 1 in Supplement 1). Need for prolonged ECMO support was stable across seasons (30.8% vs 37.3%) (eFigure 2E and eTable 1 in Supplement 1).

Across all RSV seasons, children who required IMV were younger (median [IQR] age, 3.3 [1.6-10.7] months) than those supported with NIV (median [IQR] age, 4.7 [1.9-13.4] months) or HFNC (median [IQR] age, 5.4 [2.2-13.7] months; *P* < .001) (eFigure 3A and eTable 2 in Supplement 1). Patients supported with HFNC or NIV were significantly older during the 2022-2023 postpandemic season than prepandemic (median [IQR] age for HFNC, 6.9 [2.7-16.0] months vs 4.6 [2.0-11.7] months; for NIV, 6.0 [2.1-16.5] months vs 4.3 [1.9-11.9] months) (Figure 4B and eTable 2 in Supplement 1). When stratified by age, the proportion of patients supported with HFNC significantly increased in the postpandemic period among patients aged 1 to 2 years (16.0% vs 13.2%; *P* < .001) (eTable 3 in Supplement 1) and 2 to 5 years (8.4% vs 7.1%; *P* < .001) (eTable 3 in Supplement 1) but was not significantly increased in infants younger than 1 year (16.7% vs 16.1%; *P* = .053) (eTable 3 in Supplement 1). In contrast, the age of children receiving mechanical ventilation was not different in the 2022 to 2023 postpandemic season compared with prepandemic seasons (median [IQR], 3.5 vs 3.5 months) (Figure 4B and eTable 2 in Supplement 1), and the age-stratified proportion of patients requiring IMV decreased across all age strata in the 2022 to 2023 postpandemic season (eTable 3 in Supplement 1).

Requirement for more invasive respiratory support was associated with increased prevalence of comorbid conditions (16.7% for HFNC, 25.9% for NIV, and 35.7% for IMV; *P* < .001) (eFigure 3B in Supplement 1). Notably, however, comorbid conditions were less frequent during the 2022 to 2023 postpandemic season relative to prepandemic seasons within each respiratory support category (HFNC, 14.9% vs 19.1%; NIV, 22.0% vs 28.5%; IMV, 30.5% vs 38.0%; *P* < .001) (Figure 4C and eTable 2 in Supplement 1).

## Discussion

This is the largest US database study to date examining pediatric RSV infection focused on use of advanced respiratory support modes and clinical metrics of disease severity before and after the COVID-19 pandemic. This retrospective cross-sectional study of 288 816 children 5 years or younger with RSV from 48 pediatric hospitals between 2017 and 2023 is consistent with regional and national data reporting a marked decrease in pediatric RSV infection during the COVID-19 pandemic, followed by a resurgence of RSV in 2022 to 2023^18,19,20,44^ in older and more previously healthy children.^19,21,22,23,45^ In this analysis, we report for the first time, to our knowledge, differences in the use of advanced respiratory support modes, including HFNC, during the 2022 to 2023 postpandemic season and determined differences in age and medical comorbidities that distinguished the postpandemic pediatric RSV population.

The resurgence of pediatric RSV infections in 2022 to 2023 resulted in a marked 3.5-fold higher number of children presenting to EDs than in prepandemic seasons. Although a lower proportion required hospitalization or ICU admission than typical RSV seasons, the strain on pediatric hospital systems was apparent in the 50% increase in total hospital-days and 25% increase in ICU-days during the 2022 to 2023 postpandemic season in this analysis. We report a 70.1% increase in the number of children supported with advanced respiratory support during the 2022 to 2023 postpandemic season, underscoring the burden on health care resources. Although 21.6% of children across seasons required advanced respiratory support, the increase during the 2022 to 2023 postpandemic season was largely driven by increases in use of noninvasive support with HFNC and NIV. In this cohort, the number of patients supported with HFNC and total HFNC-days nearly doubled in 2022 to 2023, whereas the proportions of children requiring IMV and total IMV-days were stable from prepandemic data. These findings align with previous studies reporting a progressive trend for increasing HFNC and NIV use in pediatric bronchiolitis with relatively consistent IMV use.^4,34,35,36,37^ Previous studies have suggested that less commonly used respiratory support modalities, including HFV, ECMO, and iNO, may be necessary for severe RSV-induced respiratory failure.^4,46,47,48,49,50,51^ We found that use of iNO decreased significantly in the postpandemic season, whereas HFV and ECMO use was unchanged. Our analysis of duration of advanced respiratory support revealed that a lower proportion of patients required prolonged support despite higher total respiratory support–days in the HFNC, NIV, and IMV cohorts. Together, these findings are consistent with a large surge in children requiring advanced respiratory support, albeit for a shorter duration per patient, during the 2022 to 2023 postpandemic season. These findings highlight concern for potential repeated outpacing of pediatric hospital and ICU resources if these trends persist through future RSV seasons.^27,37^

A unique finding in this study is that patients supported with HFNC and NIV were significantly older during the 2022 to 2023 postpandemic season than in prepandemic seasons, in contrast to patients receiving IMV, whose age was not different. Notably, HFNC was used more frequently in children aged 1 to 5 years in the 2022 to 2023 postpandemic season, whereas use of HFNC in infants younger than 1 year remained stable compared with the prepandemic period. These findings highlight a trend for increasing HFNC use among older children with RSV infection in the postpandemic era.^52^ Consistent with historical data,^4,5,6,9^ preexisting comorbid conditions were more common in children who required more invasive respiratory support. However, regardless of respiratory support mode (ie, HFNC, NIV, or IMV), the rates of comorbid conditions were lower in all categories during the 2022 to 2023 postpandemic season.^53^ In this study, we found that although the absolute number of hospital admissions and patients requiring respiratory support increased, the proportion of children with RSV requiring hospital or ICU admission and ventilatory support did not increase during the 2022 to 2023 postpandemic season. This finding could reflect an increase in the susceptible pool of children exposed to RSV for the first time after cessation of pandemic-related masking and social distancing precautions,^15,16,17^ although alterations in RSV virulence after the pandemic should also be considered.^54^ The increased rate of respiratory support needs in older, previously healthy children during the 2022 to 2023 postpandemic season aligns with the hypothesis that an “immunity debt”^17^ in children who evaded RSV infection in their first years of life during the early COVID-19 pandemic has shifted the demographics of pediatric RSV infection in the postpandemic era.^52,53^

Respiratory syncytial virus surveillance tracking suggests that postpandemic pediatric RSV surges may not be limited to the 2022 to 2023 season.^15^ For the first time in 30 years, new US Food and Drug Administration–approved RSV monoclonal antibody prophylaxis and maternal vaccines^55,56^ have created opportunities to mitigate the public health burden of RSV in vulnerable children. With limited supply of both nirsevimab and Abrysvo,^57^ the question remains, “Who should receive these prophylactic therapies?” Current American Academy of Pediatrics and Centers for Disease Control and Prevention guidelines recommend RSV monoclonal antibody prophylaxis for children younger than 8 months and for certain high-risk children aged 8 to 19 months.^58^ This study revealed a substantial burden on pediatric hospital systems during the 2022 to 2023 RSV season in previously healthy, older children who required advanced respiratory support. As we look ahead to future postpandemic RSV seasons, data on respiratory support mode use for pediatric RSV infections can help guide RSV prophylaxis eligibility guidelines for distribution of limited supply in vulnerable children.

### Limitations

This study has limitations. We included 48 pediatric hospitals available in the PHIS database; therefore, the study population may not represent the entire US pediatric population. Most hospitals reporting to the PHIS database are large university medical centers; therefore, this study lacks perspectives of smaller hospitals and children’s hospitals integrated within adult hospitals. Hospitals in the PHIS database are heterogeneously distributed across the US, and certain regions may be disproportionately represented in the dataset. *ICD-10* codes were used to identify patients with RSV, which could overestimate or underestimate RSV infections if patients were not tested or if testing was done at an outside hospital.^59,60^ Viral and bacterial coinfections impact RSV severity, but these data are not available. Viral respiratory testing has become more widely available in the postpandemic period, which could impact increases in the total number of patients with RSV in this study. Hospital and physician practice variations in respiratory support mode use for RSV infection is a confounder that could not be controlled for in this analysis. To date, PHIS studies have not generally reported HFNC use due to variations in billing practice.^40^ Recent updates and remapping of PHIS have allowed for reliable reporting of HFNC but are limited to 33 of the 48 PHIS hospitals, which is a limitation of our analysis. Although consistent across seasons, the sample size for rare respiratory support modes, including ECMO, HFV, and iNO, is small. Limitations of the PHIS database precluded accurately identifying patients requiring only supplemental oxygen, preventing analysis of more mild RSV disease. We acknowledge the challenges of making epidemiologic and systems-based conclusions during an infectious disease surge given inherent alterations in typical resource allocation, hospital transfers, and internal inpatient and ICU admission criteria rationale.

## Conclusions

In this retrospective cross-sectional study, we report a postpandemic pediatric RSV surge with significant increases in ED presentations, total hospital- and ICU-days, and number of children requiring advanced respiratory support. Notably, noninvasive respiratory support with HFNC and NIV was used more frequently during the 2022 to 2023 postpandemic season than in previous seasons, whereas mechanical ventilation use was similar. Moreover, the postpandemic pediatric RSV population requiring respiratory support was older with fewer comorbid conditions than in the prepandemic period. The postpandemic surge of RSV in both vulnerable, younger populations and older, previously healthy children led to substantial increases in US hospital volumes and health care system burden. Although these trends need to be evaluated in subsequent years, this study highlights possible epidemiologic shifts and trends in respiratory support use that may help inform guidelines and expanded age considerations for new RSV vaccines as they become more widely available.

## Notes

Supplement 1. **eTable 1.** Patient Characteristics by All RSV Seasons **eTable 2.** Age and Comorbidities Across RSV Seasons **eTable 3.** Respiratory Support Requirements Stratified by Age **eFigure 1.** Mortality and CPR Rates by RSV Season **eFigure 2.** Respiratory Support Rates and Duration of Therapy by Season **eFigure 3.** Age and Comorbid Conditions for Patients Requiring Respiratory Support for All RSV Seasons

Supplement 2.
Data Sharing Statement
