# Supplementary material for: Pediatric Respiratory Syncytial Virus Hospitalizations and Respiratory Support After the COVID-19 Pandemic
Source: JAMA Netw Open. 2024 Jun 13;7(6):e2416852. doi: 10.1001/jamanetworkopen.2024.16852 (PMC11177168; doi:10.1001/jamanetworkopen.2024.16852)
Supplement: Supplement 1. — eTable 1. Patient Characteristics by All RSV Seasons eTable 2. Age and Comorbidities Across RSV Seasons eTable 3. Respiratory Support Requirements Stratified by Age eFigure 1. Mortality and CPR Rates by RSV Season eFigure 2. Respiratory Support Rates and Duration of Therapy by Season eFigure 3. Age and Comorbid Conditions for Patients Requiring Respiratory Support for All RSV Seasons [file jamanetwopen-e2416852-s001.pdf]

Supplementary Online Content

Winthrop ZA, Perez JM, Staffa SJ, McManus ML, Duvall MG. Pediatric respiratory syncytial virus hospitalizations and respiratory support after the COVID-19 pandemic. *JAMA Netw Open*. 2024;7(6):e2416852. doi:10.1001/jamanetworkopen.2024.16852

eTable 1. Patient Characteristics by All RSV Seasons

eTable 2. Age and Comorbidities across RSV Seasons

eTable 3. Respiratory Support Requirements Stratified by Age

eFigure 1. Mortality and CPR rates by RSV Season

eFigure 2. Respiratory Support Rates and Duration of Therapy by Season

eFigure 3. Age and Comorbid Conditions for Patients Requiring Respiratory Support for All

RSV Seasons

This supplementary material has been provided by the authors to give readers additional information about their work.

© 2024 Winthrop ZA et al. *JAMA Network Open.*

eTable 1. Patient Characteristics by All RSV Seasons

Characteristic	Overall 2017-2023 (n = 288,816)	2017-2018 (n = 34,834)	2018-2019 (n = 37,670)	2019-2020 (n = 46,591)	Prepandemic (2017-2020 mean)[a] (n = 119,095; mean n = 39,698)	2020-2021 (n = 6,985)	2021-2022 (n = 68,389)	Postpandemic (2022-2023 Season) (n = 94,347)	P value[b]
**Age, median (IQR), mo**									
ED and Inpatient	8.9 (3.3, 21.5)	6.2 (2.4, 15.8)	6.7 (2.6, 16.7)	7.4 (2.9, 17.6)	6.8 (2.6, 16.8)	11.5 (4.3, 23.3)	10 (3.6, 23.2)	11.3 (4.1, 26.6)	
ED	11.5 (5, 24.8)	7.4 (3.5, 15.6)	8.4 (3.8, 17.8)	9.4 (4.3, 19.5)	8.5 (3.9, 17.9)	14.3 (6.2, 25.8)	13.2 (5.4, 26.8)	13.7 (5.8, 29.8)	<0.001
Inpatient	7.2 (2.5, 19.0)	5.7 (2, 15.9)	6 (2.1, 16.2)	6.3 (2.3, 16.3)	6 (2.2, 16.1)	9.5 (3.4, 21.4)	7.6 (2.6, 20.1)	9 (3.1, 23.4)	
**Age group, mo**									
0-12	167417 (58.0)	23505 (67.5)	24584 (65.3)	29521 (63.4)	25870 (65.2)	3572 (51.1)	37460 (54.8)	48775 (51.7)	
12-24	57942 (20.1)	6196 (17.8)	7120 (18.9)	9169 (19.7)	7495 (18.9)	1751 (25.1)	14529 (21.2)	19177 (20.3)	<0.001
≥24-60	63457 (22)	5133 (14.7)	5966 (15.8)	7901 (17)	6332 (16.0)	1662 (23.8)	16400 (24.0)	26395 (28.0)	
**Sex**									
Female	129403 (44.8)	15398 (44.2)	16801 (44.6)	20778 (44.6)	17658 (44.5)	3144 (45.0)	30821 (45.1)	42461 (45.0)	
Male	159348 (55.2)	19418 (55.7)	20850 (55.4)	25808 (55.4)	22024 (55.5)	3841 (55.0)	37561 (54.9)	51870 (55.0)	0.002
Unknown	65 (0.02)	18 (0.05)	19 (0.05)	5 (0.01)	16 (0.04)	0 (0.0)	7 (0.01)	16 (0.02)	
**Race and Ethnicity**									
Asian or Pacific Islander	8076 (2.8)	948 (2.7)	1032 (2.7)	1187 (2.6)	1056 (2.7)	85 (1.2)	1648 (2.4)	3176 (3.4)	<0.001
Hispanic	73220 (25.4)	8946 (25.7)	8778 (23.3)	10932 (23.5)	9552 (24.1)	1817 (26)	17585 (25.7)	25162 (26.7)	
Multiracial	5323 (1.8)	425 (1.2)	500 (1.3)	751 (1.6)	559 (1.4)	113 (1.6)	1291 (1.9)	2243 (2.4)	
Non-hispanic Black	54551 (18.9)	6056 (17.4)	6784 (18.0)	8632 (18.5)	7157 (18.0)	1859 (26.6)	14010 (20.5)	17210 (18.2)	
Non-hispanic White	121953 (42.2)	15042 (43.2)	16751 (44.5)	20851 (44.8)	17548 (44.2)	2782 (39.8)	28316 (41.4)	38211 (40.5)	
Other and Unknown[c]	25693 (8.9)	3417 (9.8)	3825 (10.2)	4238 (9.1)	3826 (9.6)	329 (4.7)	5539 (8.1)	8345 (8.9)	
**Child Opportunity Index Score, median (IQR)**	45 (21, 72) n=288324	43 (20, 69) n=34770	44 (20, 71) n=37596	44 (21, 70) n=46487	44 (20, 70) n=118853	40 (18, 64) n=6970	46 (22, 72) n=68287	47 (22, 74) n=94214	<0.001
**Level of Service**									
Emergency Department	114421 (39.6)	9902 (28.4)	11979 (31.8)	15871 (34.1)	12584 (31.7)	2786 (39.9)	30155 (44.1)	43728 (46.4)	
Inpatient	174395 (60.4)	24932 (71.6)	25691 (68.2)	30720 (65.9)	27114 (68.3)	4199 (60.1)	38234 (55.9)	50619 (53.6)	<0.001
ICU and NICU (% of Inpatient)	54053/174395 (30.9)	8720/24932 (35.0)	9283/25691 (36.1)	10636/30720 (34.6)	9546/27114 (35.2)	1142/4199 (27.2)	10576/38234 (27.7)	13696/50619 (27.1)	
**Complex chronic conditions (% of inpatients and observation patients)**	34209/174395 (19.6)	5797/24932 (23.3)	5645/25691 (22)	6287/30720 (20.5)	5910/27114 (21.8)	771/4199 (18.4)	6784/38234 (17.7)	8925/50619 (17.6)	<0.001
**Length of inpatient stay, median (IQR), days**									
Hospital stay	3 (1, 5)	3 (2, 5)	3 (2, 5)	3 (2, 5)	3 (2, 5)	2 (1, 4)	2 (1, 4)	3 (1, 4)	<0.001
ICU and NICU stay	3 (2, 5)	3 (2, 6)	3 (2, 6)	3 (2, 5)	3 (2, 6)	2 (1, 4)	3 (2, 5)	3 (2, 5)	
**Total Hospital and ICU days**									
Hospital days	798095	136013	133419	147806	139,079	19712	149537	211608	NA
ICU days	299283	55733	56122	59509	57,121	7305	50062	70552	
**Respiratory Support (% of Inpatients)**									
HFNC[d]	19002/136497 (13.9)	2737/18952 (14.4)	2712/19321 (14.0)	3387/24089 (14.1)	2945/20787 (14.2)	470/3722 (12.6)	3944/30787 (12.8)	5752/39626 (14.5)	0.297
NIV	7800/174395 (4.5)	1318/24932 (5.3)	1384/25691 (5.4)	1536/30720 (5)	1413/27114 (5.2)	79/4199 (1.9)	1404/38234 (3.7)	2079/50619 (4.1)	<0.001
IMV	4982/174395 (2.9)	978/24932 (3.9)	932/25691 (3.6)	1037/30720 (3.4)	982/27114 (3.6)	44/4199 (1.1)	728/38234 (1.9)	1263/50619 (2.5)	<0.001
HFV	73/174395 (0.04)	16/24932 (0.06)	15/25691 (0.06)	25/30720 (0.08)	19/27114 (0.07)	1/4199 (0.02)	9/38234 (0.02)	7/50619 (0.01)	<0.001
ECMO	48/174395 (0.03)	8/24932 (0.03)	9/25691 (0.04)	10/30720 (0.03)	9/27114 (0.03)	0/4199 (0)	8/38234 (0.02)	13/50619 (0.03)	0.303
iNO	383/174395 (0.22)	95/24932 (0.38)	102/25691 (0.4)	97/30720 (0.32)	98/27114 (0.36)	4/4199 (0.1)	33/38234 (0.09)	52/50619 (0.10)	<0.001
**Total Respiratory Support Days, No.**									
HFNC	54667	8292	8157	10180	8876	1217	10898	15923	NA
NIV	24224	4456	4487	4894	4612	278	3995	6114	
IMV	28000	6069	5253	6413	5912	531	3630	6104	
ECMO	456	76	100	72	83	0	79	129	
**Mortality (% of Inpatient and ICU patients)**									
Inpatient and Observation	294/174395 (0.17)	52/24932 (0.21)	46/25691 (0.18)	61/30720 (0.20)	53/27114 (0.2)	9/4199 (0.21)	58/38234 (0.15)	68/50619 (0.13)	0.009
ICU and NICU	282/54053 (0.52)	51/8720 (0.59)	44/9283 (0.55)	59/10636 (0.56)	51/9546 (0.53)	9/1142 (0.79)	55/10576 (0.52)	64/13696 (0.47)	0.328
**CPR (% of Inpatient)**									
Inpatient and Observation	353/174395 (0.20)	75/24932 (0.30)	78/25691 (0.30)	77/30720 (0.25)	77/27114 (0.28)	0/4199 (0.0)	58/38234 (0.15)	65/50619 (0.13)	<0.001
ICU and NICU	279/54053 (0.52)	64/8720 (0.73)	66/9283 (0.71)	63/10636 (0.59)	64/9546 (0.67)	0/1142 (0.0)	42/10576 (0.40)	44/13696 (0.32)	<0.001

© 2024 Winthrop ZA et al. *JAMA Network Open.*

Abbreviations: ED, emergency department; ICU, intensive care unit; NICU, neonatal intensive care unit; CCC, complex chronic condition; HFNC, high-flow nasal cannula; NIV, noninvasive ventilation; IMV, invasive mechanical ventilation; HFV, high-frequency ventilation; ECMO, extracorporeal membrane oxygenation; iNO, inhaled nitric oxide.

a. Pre-pandemic seasons data is reported as averaged data including the 2017-18, 2018-19, and 2019-20 seasons. Percentages are reported as the frequency amongst all patients (% of total; including ED and hospitalized patients), or as the frequency of hospitalized patients (% of inpatient).
b. Kruskal-Wallis test was used for continuous variables and Pearson X^2 test and Cochran-Armitage Chi-square test for trend for categorical variables to assess for statistical differences. Comparisons between pre-pandemic and post-pandemic RSV seasons was performed using the Wilcoxon rank sum test. $p < 0.05$ was considered statistically significant.
c. Includes patients denoted as “Other” in the Pediatric Health Information System database and those who declined to provide race and ethnicity information.
d. HFNC data was only available from 33 of the 48 study hospitals, therefore the denominator is different from other respiratory support types. Proportions were calculated based on % of inpatients admitted to those 33 hospitals.

© 2024 Winthrop ZA et al. *JAMA Network Open.*

eTable 2. Age and Comorbidities across RSV Seasons

	2017-18	2018-19	2019-20	Prepandemic (2017-2020 Mean)[a] (n = 81343)	2020-21	2021-22	Postpandemic (2022-2023 Season) (n = 50619)	P value[b]
**Age by Respiratory Support, median (IQR), mo**								
HFNC	4.7 (2, 12)	4.4 (1.9, 11.5)	4.6 (2, 11.6)	4.6 (2, 11.7)	6.4 (2.9, 15.4)	5.4 (2.3, 14.5)	6.9 (2.7, 16)	<0.001
NIV	4.4 (1.9, 12.4)	4.5 (1.9, 12.5)	4.2 (1.9, 11)	4.3 (1.9, 11.9)	3.7 (1.7, 8.2)	4.5 (1.8, 13.5)	6 (2.1, 16.5)	<0.001
IMV	3.9 (1.7, 12.2)	3.6 (1.7, 11.7)	3 (1.5, 9.2)	3.5 (1.6, 10.8)	2.5 (1.4, 14.3)	2.7 (1.4, 8.6)	3.5 (1.6, 12.5)	0.594
ECMO	4 (3.4, 18.3)	5.6 (3.6, 7.5)	4.5 (3, 9.7)	4.2 (3, 9.7)	n=0	12.5 (2.9, 14.5)	9.5 (4, 16.5)	0.378
**Comorbidity by Respiratory Support (%CCC of total)**								
HFNC	605/2737 (22.1)	473/2712 (17.4)	610/3387 (18)	1688/8836 (19.1)	55/470 (11.7)	572/3944 (14.5)	859/5752 (14.9)	<0.001
NIV	405/1318 (30.7)	394/1384 (28.5)	408/1536 (26.6)	1207/4238 (28.5)	24/79 (30.4)	330/1404 (23.5)	457/2079 (22)	<0.001
IMV	396/978 (40.5)	337/932 (36.2)	388/1037 (37.4)	1121/2947 (38)	20/44 (45.5)	254/728 (34.9)	385/1263 (30.5)	<0.001
ECMO	7/8 (87.5)	7/9 (77.8)	9/10 (90)	23/27 (85.2)	n=0	7/8 (87.5)	10/13 (76.9)	0.525

Abbreviations: ED, emergency department; ICU, intensive care unit; NICU, neonatal intensive care unit; CCC, complex chronic condition; HFNC, high-flow nasal cannula; NIV, noninvasive ventilation; IMV, invasive mechanical ventilation; ECMO, extracorporeal membrane oxygenation.

a. Pre-pandemic seasons data is reported as averaged data including the 2017-18, 2018-19, and 2019-20 seasons. Percentages are reported as the frequency amongst all patients (% of total, including ED and hospitalized patients), or as the frequency of hospitalized patients (% of inpatient).
b. P values were calculated using Wilcoxon rank sum test. $p < 0.05$ was considered statistically significant.

© 2024 Winthrop ZA et al. *JAMA Network Open.*

eTable 3. Respiratory Support Requirements Stratified by Age

Respiratory Support (% of Inpatient)	Prepandemic (2017-2020 mean)[a]	Postpandemic (2022-2023 Season)	P value[b]
	Age Strata: 0 to <1 years		
Required HFNC[c]	2227/13804 (16.1)	3739/22353 (16.7)	0.053
Required NIV	1062/18121 (5.9)	1376/28813 (4.8)	<0.001*
Required IMV	753/18121 (4.2)	941/28813 (3.3)	<0.001*
	Age Strata: 1 to <2 years		
Required HFNC	486/3689 (13.2)	1192/7455 (16)	<0.001*
Required NIV	210/4750 (4.4)	373/9428 (4)	0.078
Required IMV	132/4750 (2.8)	153/9428 (1.6)	<0.001*
	Age Strata: 2 to 5 years		
Required HFNC	232/3294 (7.1)	821/9818 (8.4)	0.001*
Required NIV	141/4243 (3.3)	330/12378 (2.7)	0.003*
Required IMV	97/4243 (2.3)	169/12378 (1.4)	<0.001*

Abbreviations: HFNC, high-flow nasal cannula; NIV, noninvasive ventilation; IMV, invasive mechanical ventilation;

a. Pre-pandemic seasons data is reported as averaged data including the 2017-18, 2018-19, and 2019-20 seasons. Percentages are reported as the frequency amongst all patients (% of total, including ED and hospitalized patients), or as the frequency of hospitalized patients (% of inpatient).
b. Kruskal-Wallis test was used for continuous variables and Pearson X^2 test and Cochran-Armitage Chi-square test for trend for categorical variables to assess for statistical differences. Comparisons between pre-pandemic and post-pandemic RSV seasons was performed using the Wilcoxon rank sum test. $p < 0.05$ was considered statistically significant.
e. HFNC data was only available from 33 of the 48 study hospitals, therefore the denominator is different from other respiratory support types. Proportions were calculated based on % of inpatients admitted to those 33 hospitals.

© 2024 Winthrop ZA et al. *JAMA Network Open.*

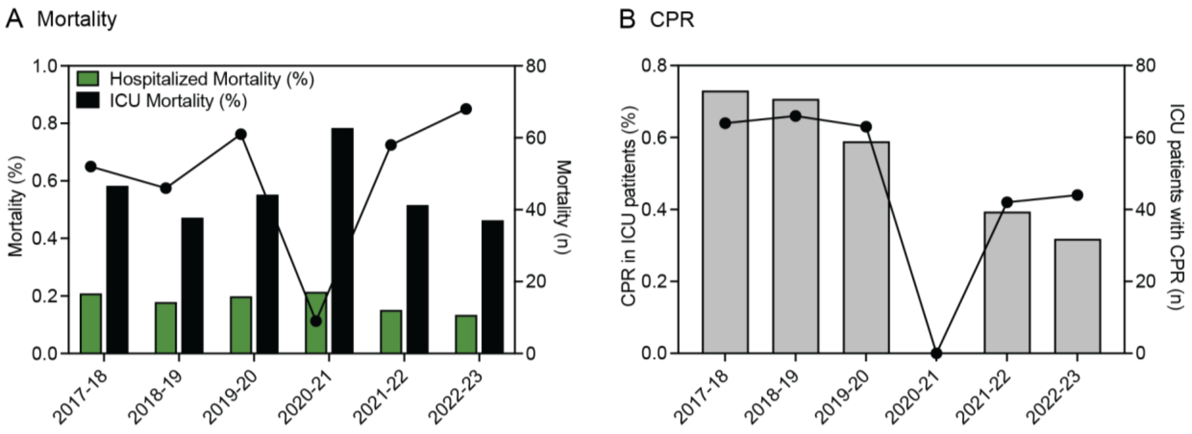

eFigure 1. Mortality and CPR rates by RSV Season.

Total number and proportional Hospital and ICU mortality (A), total number and proportional ICU CPR (B). RSV seasons (July 1 to June 30) are noted on the X axis. Bars (left y-axis) indicate the proportion of total hospitalized or ICU patients. Circles with connecting line (right y-axis) denotes the total number of patients. Each panel has a different y-axis scale to show trends. Abbreviations: CPR, cardiopulmonary resuscitation.

© 2024 Winthrop ZA et al. *JAMA Network Open.*

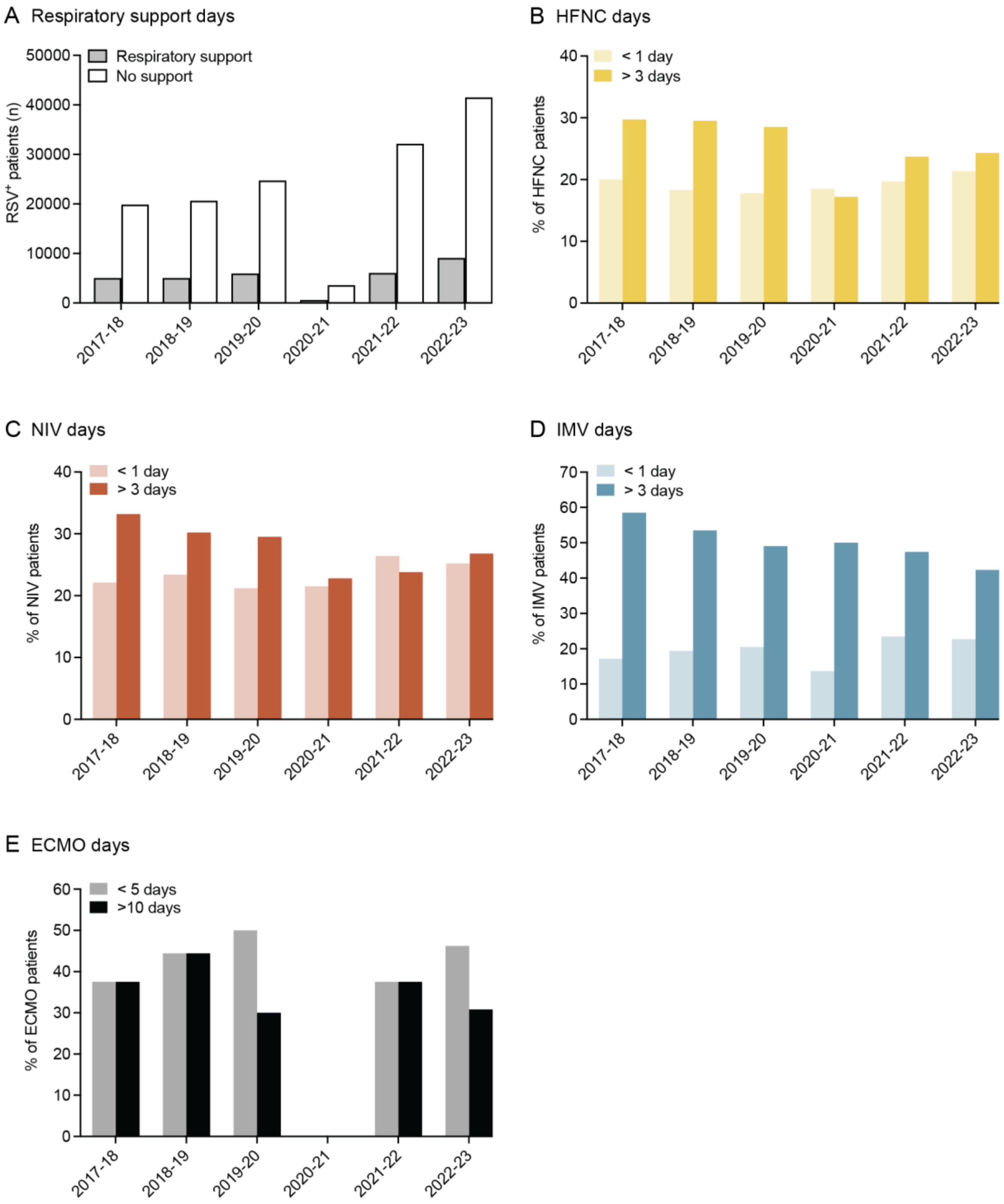

eFigure 2. Respiratory Support Rates and Duration of Therapy by Season.
Number of patients who required any respiratory support modality or did not require advanced respiratory support (A), proportion of patients supported with HFNC for <1 day or >3 days (B), proportion of patients supported with NIV for <1 day or >3 days (C), proportion of patients supported with IMV for <1 day or >3 days (D), proportion of patients who required ECMO supported for <5 days or > 10 days (E). RSV season is denoted on the x-axis. Abbreviations: HFNC, high-flow nasal cannula; NIV, noninvasive ventilation; IMV, invasive mechanical ventilation; ECMO, extracorporeal membrane oxygenation.

© 2024 Winthrop ZA et al. *JAMA Network Open.*

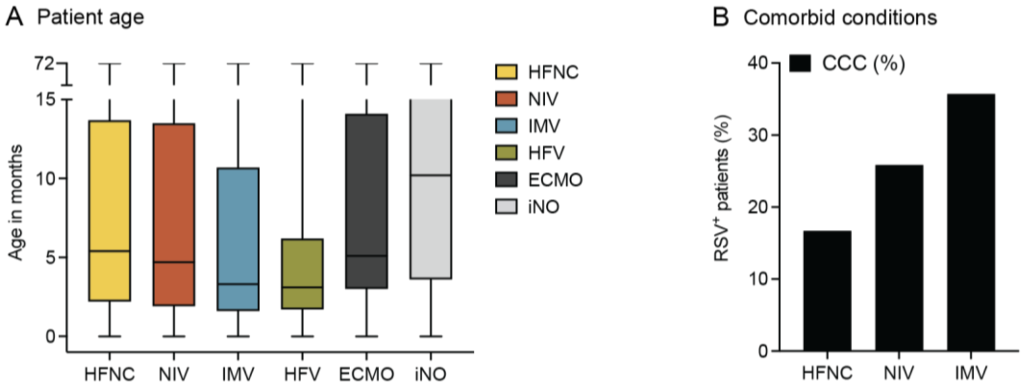

eFigure 3. Age and Comorbid Conditions for Patients Requiring Respiratory Support for All RSV Seasons Combined.

Bars show age in months (median, IQR, range) for entire study population by respiratory support types (A), proportion of patients with comorbid conditions for entire study population by respiratory support type (B). X-axis represents the collective population of patients requiring varying respiratory support modalities from all 6 RSV seasons combined from July 2017 through June 2023. Abbreviations: HFNC, high-flow nasal cannula; NIV, noninvasive ventilation; IMV, invasive mechanical ventilation; HFV, high-frequency ventilation; ECMO, extracorporeal membrane oxygenation; iNO, inhaled nitric oxide.

© 2024 Winthrop ZA et al. *JAMA Network Open.*
