# Supplementary material for: Pediatric Respiratory Syncytial Virus Hospitalizations and Respiratory Support After the COVID-19 Pandemic
Source: JAMA Netw Open. 2024 Jun 13;7(6):e2416852. doi: 10.1001/jamanetworkopen.2024.16852 (PMC11177168; doi:10.1001/jamanetworkopen.2024.16852)
Supplement: Supplement 2. — Data Sharing Statement [file jamanetwopen-e2416852-s002.pdf]

Data Sharing Statement

Winthrop. Pediatric Respiratory Syncytial Virus Hospitalizations and Respiratory Support After the COVID-19 Pandemic. *JAMA Netw Open*. Published June 13, 2024.
doi:10.1001/jamanetworkopen.2024.16852

Data
Data available: No

Additional Information
Explanation for why data not available: The data for this study is owned by the Children's Hospital Association. Any hospital may join at:
https://www.childrenshospitals.org/aboutcha/about/membership
